# Exploring Pediatric Nurses’ Perspectives on Their Work Environment, Work Attitudes, and Experience of Burnout: What Really Matters?

**DOI:** 10.3389/fped.2022.851001

**Published:** 2022-03-17

**Authors:** Laura Buckley, Whitney Berta, Kristin Cleverley, Kimberley Widger

**Affiliations:** ^1^Lawrence S. Bloomberg Faculty of Nursing, University of Toronto, Toronto, ON, Canada; ^2^The Hospital for Sick Children, Toronto, ON, Canada; ^3^Institute of Health Policy, Management and Evaluation, University of Toronto, Toronto, ON, Canada; ^4^The Margaret and Wallace McCain Centre for Child, Youth and Family Mental Health, Centre for Addiction and Mental Health, Toronto, ON, Canada

**Keywords:** burnout, quality of work-life, work engagement, nurses, pediatric, qualitative

## Abstract

**Background:**

Pediatric nurses care for some of the most vulnerable patients in our healthcare system and are vulnerable to the impact of the stress of their work on their well-being. Burnout is a potential response to chronic interpersonal stressors and a negative work outcome linked to personal and professional consequences. A thorough understanding of the experience and factors associated with burnout in this population is an important part of developing interventions to mitigate or prevent this workplace outcome. Therefore, our study objectives were to: (1) explain and expand our understanding of pediatric critical care nurses experience of burnout in relation to their work environment and work engagement; (2) provide recommendations for nursing administrators to improve nurses’ work environment, work attitudes, and work outcomes.

**Methods:**

A convenience sample of pediatric critical care nurses from a large pediatric quaternary care hospital in Ontario, Canada were invited to participate in this second phase of a sequential explanatory mixed-methods study. Semi-structured interviews were conducted, with and main themes and subthemes distilled through the method of interpretive description.

**Results:**

A total of 18 PICU/CCCU/NICU nurses participated. Derived themes included the experience and identification of burnout, including its prevalence and elusiveness. Their experiences of quality of work-life included themes such as compensation, emotional support at work, respect, their professional identity, and spill over into home life. They discussed components of work engagement, including the work itself, investment into their growth and development, and the meaning of their work. The self-care subthemes included the importance of preparation and recovery, and the use of physical and mental separation as a preservation strategy. The participants’ recommendations for strategies to mitigate burnout were also summarized.

**Conclusion:**

Burnout is a complex and regularly occurring experience for pediatric critical care nurses. Although the experience may be difficult to self-identify, the impacts on the individuals are profound. Further research and organizational support are needed to test practical and evidence-based interventions to improve the well-being of this population.

## Introduction

Even prior to the SARS-CoV-2 (COVID-19) pandemic, frontline critical care workers were known to be on the brink of a well-being crisis (1–3). There are multiple sources of distress in critical care, including clinical situations involving end-of-life or prolongation of life, contextual factors related to the work environment, team communication and relationships, and more recently, resource related dilemmas (4). In 2014, many organizations added a fourth objective to the Institute for Health Care Improvement’s Triple Aim of Health Care focused on health care provider well-being, in acknowledgment of the impact provider well-being has on patient satisfaction, clinical outcomes, and health care costs (5). In 2016, the Critical Care Societies Collaborative (CCSC) based in the United States, published “A Call for Action” on burnout in critical care health care professionals urging key stakeholders to address critical care health care provider burnout to improve both patient and provider well-being (6). Locally, Critical Care Services Ontario (CCSO) has recently begun publishing province-wide surveys of critical care practitioner burnout to monitor the well-being of Ontario’s critical care staff (7).

Burnout is a psychological syndrome emerging as a prolonged response to chronic interpersonal stressors on the job (8). Burnout has shown to have physical, psychological and occupational consequences across working populations (9). Maslach states that there are three key dimensions of burnout: emotional exhaustion, depersonalization, and lack of accomplishment (8). When applied to health care settings, emotional exhaustion refers to when the health care provider feels emotionally drained from their work. Depersonalization is the development of cynicism, particularly toward patients. Lack of personal accomplishment is when the health care provider feels a sense of ineffectiveness and dissatisfaction with the care they are providing (10). Burnout impacts at the level of the provider, the patient, and the organization (11).

While all health care providers are at high risk of burnout, pediatric nurses care for some of the most vulnerable patients in our healthcare system and are particularly vulnerable to the impact of the stress of their work on their well-being. These nurses skillfully manage the highly specialized care of children and the complex family dynamics that are inherent to the work (11). A recent scoping review demonstrated that burnout was prevalent in pediatric nurses and was related to aspects of the work environment, work attitudes, and work outcomes (11).

Within the population of pediatric nurses, pediatric/neonatal critical care nurses are a subspecialty within a specialty. These highly specialized nurses cannot be easily replaced or supplemented and they care for the most severely ill and injured children at the highest risk of death (12, 13). In 2021, we used the Theory of Reasoned Action to guide an examination of modifiable work environment factors that had the greatest association with the work outcome of burnout in a sample of pediatric/neonatal critical care nurses. Simplified, the Theory of Reasoned Action states that our beliefs about our work environment, influence our work attitudes and are directly related to our behavioral intentions and, in turn, our behaviors at work (work outcomes) (14–16). We conducted a survey to examine factors of the work environment (e.g., quality of work-life, perceived organizational support, and workplace incivility) and work attitudes (e.g., work engagement) of pediatric critical care nurses and their relationship to burnout. Quality of work-life and work engagement were identified as the most important factors (17). However, in the interests of mitigating or preventing burnout, a thorough understanding of the experience of workplace burnout and related factors from the perspective of nurses is required. Therefore, building on our survey findings we aimed to: (1) explain and expand our understanding of pediatric critical care nurses experience of burnout in relation to their work environment and work engagement and (2) provide recommendations for nursing administrators to improve nurses’ work environment, work attitudes, and work outcomes.

## Materials and Methods

### Study Design

This study is the second phase of a two-phased mixed methods evaluation of pediatric nurses working in critical care using a convenience sample of nurses at a large quaternary care pediatric hospital in Toronto, Canada. The explanatory sequential mixed methods design began with a quantitative cross-sectional survey which was developed based on the Theory of Reasoned Action (17). Study findings were used to build the semi-structured interview guide for the current study.

Guided by the methodology of constructivist grounded theory, and using the analytic method of interpretive description, the current study utilized semi-structured qualitative interviews to contextualize, illuminate, and explore the experience of burnout and related factors at work in the sample of pediatric critical care nurses (18).

### Study Location and Context

The hospital is a 300-bed tertiary care hospital with a 41-bed critical care unit and 36-bed Neonatal Intensive Care Unit (NICU). The critical care unit is divided into multi-organ medical-surgical, and cardiac services. Four-hundred and forty-three RNs work in the combined Cardiac Critical Care Unit (CCCU), Pediatric Intensive Care Unit (PICU), and NICU. These units are largely homogeneous in the nurses’ skill set and acuity of their work here, and across similar facilities, which increases transferability of the results (19). Interviews were conducted in the Summer of 2021 during the COVID-19 pandemic. Of note, in an unprecedented move, the PICU admitted adult COVID-19 patients in the months leading up to the interviews which was a unique experience for the pediatric nurses working this time. The adult patients had been discharged 3 weeks prior to initiation of data collection.

### Eligibility and Recruitment

Registered Nurses who had worked in the PICU, CCCU, or NICU for more than 3 months and completed a survey in the quantitative phase of the study were eligible to take part in the study. Nurses who participated in the quantitative phase of the study were asked to indicate if they were interested in participating in the this qualitative phase of the study. Interested participants were contacted via email and semi-structured interviews were booked on a first-come-first-served bases.

### Semi-Structured Interview Guide Creation

The semi-structured interview guide was developed using the process of integration and building as described by Creswell and Creswell (19) based on the relationships uncovered in quantitative phase of the study (17). In accordance with the Theory of Reasoned Action, factors of the work environment influenced work attitudes, and work attitudes influenced work outcomes. Results of the quantitative phase indicated that quality of work-life and work engagement had the greatest impact on burnout (17). Therefore, interview guide questions were designed to more deeply explore these topics with the nurses. The interview questions were reviewed with three critical care nurses for refinement and to ensure question clarity (18). Demographic data of the participants were collected at the start of the interview. The semi-structured interview guide can be found in Supplementary Appendix A.

### Data Collection

One-on-one interviews were conducted over Zoom. Video conference allowed for flexible timing of interviews and compliance with COVID-19 social distancing rules. Interviews ranged from 25 to 60 min in length. Two trained research team members who worked in the critical care areas conducted interviews [one Clinical Nurse Specialist (LB) and one Social Worker (SS)]. However, the person who conducted a particular interview was chosen to avoid interviewing any participants who directly reported to them to promote participant comfort, participant psychological safety, and limit investigator bias. Consent was obtained from each participant prior to the commencement of the interviews. All interviews were audio recorded and subsequently transcribed. Transcriptions were reviewed by a study team member for accuracy.

### Data Analysis

Data collection and analysis were iterative allowing for clarification of concepts in subsequent interviews and increased credibility and confirmability of the results. Using the principles of interpretive description, the initial analysis of the transcripts was used to break the data down into broad themes and sub-themes in the early stages while avoiding restricting findings to the self-evident (20). Care was taken to not lose the contextual whole of the data through this process, in line with the principles of interpretive description (20, 21). Following the process described by Thorne (21), coding proceeded through intellectual inquiry (as opposed to line-by-line coding) to ensure context was respected, with the intention of constructing truths. In order to fulfill Objective 2, recommendations brought forward by the participants for nursing administrators to improve nurses’ work environment, work attitudes, and work outcomes were summarized and collated. As per the process described by Bowen et al. (18), a second team member (KW) reviewed the coding, and conflicts were discussed with a third team member (KC or WB) (18, 23). Interviews continued on a rolling basis until new themes were no longer surfacing.

## Results

### Demographic Information

A total of 18 nurses participated in the semi-structured interviews. All participants contacted for an interview consented and participated in the process. Participants came from each of the three units, four from PICU, five from CCCU, and nine from NICU. Participants of different years of experience were all represented with 8 with 0–5 years, 5 with 6–10 years, and 5 with >10 years of experience. All participants had achieved a bachelor’s degree or higher, and almost all had cared for a COVID-19 positive patient/patient under investigation. No participants worked casually, and almost all worked full-time (>0.8 FTE).

### Themes

We identified four key themes from participants’ exploration of their experiences with workplace burnout: the experience and identification of burnout, quality of work-life, work engagement and self-care. Nurses spoke about the prevalence of burnout and the challenge of identifying it within oneself. The theme of quality of work-life captures the collegial and compensatory factors that influence the nurses’ experience. The theme of work engagement includes factors of the physical work, as well as the opportunities and meaning it creates. The theme of self-care addresses the personal strategies implemented to try and mitigate their experience of burnout. Of note, self-care was not specifically explored through questions in the interview guide but came up as an important factor related to burnout across most of the interviews. Each theme and its associate subthemes are described below. All sub-themes were novel and derived exclusively from the data.

#### The Experience and Identification of Burnout

Within the theme of the experience and identification of burnout, two subthemes were identified: prevalence and elusiveness.

##### Prevalence

All participants made it evident that burnout was prevalent, and that they had experienced it at some point in their career. One participant described their experience as the following; “…*it was a lot of dread coming into work and, even once I’m there, just not wanting to be there. I think once I got home, it was just not wanting do anything and just recover*…” ***(Participant 7, NICU)***.

##### Elusiveness

Many participants discussed how burnout is highly elusive and difficult to self-identify. Participants stated; “… *you don’t know you’re burnt out until you’re like, ‘I don’t understand why I’m completely overwhelmed and I can’t do my job,’ and then (you realize), oh I’m burnt out, I didn’t know that” **(Participant 8, NICU)***. Another participant reported; “…*you have to recognize it yourself and usually people can’t” **(Participant 6, NICU)***. Another spoke about not truly understanding what burnout was until they experienced it themselves, *“I didn’t think I would burn out. I kind of didn’t think that burnout was a real thing necessarily. I thought people just got tired and needed vacations or needed a job change, which I now see are all evidence of burnout. There it is, that’s what they’ve been talking about*” ***(Participant 13, PICU)***.

#### Quality of Work-Life

Within the theme of quality of work-life five subthemes were identified: benefits and compensation; context-based emotional support; respect as a professional; identity; and quality of work-life spillover.

##### Benefits and Compensation

Benefits and compensation were frequently referenced by participants. The ability to self-schedule was by far the most positive benefit highlighted. Participants had mixed opinions on the quality and quantity of the health benefits provided. In terms of compensation, many participants cited that at a unit/organizational level, they do not get properly compensated for the length of time that they work (staying late, missing breaks), as well as feeling they should be compensated differently than ward nurses due to the nature and complexity of critical care work. “…. *working in a PICU or a NICU or the CCCU*…*I feel that the work is so much more stressful on a different level, not to say that people that work on the floor don’t have stress, but these nurses are being trained and qualified to work in an ICU. I feel that we should be compensated a little bit more*…” ***(Participant 11, NICU)***. Others brought up the broader issue of the insufficient compensation for nurses at the government level*; “*… *it does seem like a slap in the face from the government*…*we can’t get the same increases we see from police*…*.We’re also doing a very demanding job, and it would be nice if we could also see the same kind of increase that other professions get; I think that could be better” **(Participant 15, CCCU)***.

##### Context-Based Emotional Support

Participants resoundingly echoed the importance of the team to mitigate burnout. When asked what kept her coming back to work every day, one participant said, *“The people. It’s no questions asked, we have the best team. I think just the supportive nature of the relationships largely, just like-minded people that even in the worst of times understand what you do and can find lightheartedness in the worst moments. Just good people who share the passion” **(Participant 12, CCCU)***. When it comes to dealing with the specific challenges of the work many participants brought up the idea of context-based emotional support that they can only get from others who have had similar experiences. “… *(it) fills your cup with that space to vent in a way, where people understand what you’re talking about you know you can’t go and talk to your non-nursing friends about things, you can’t go to therapy and talk about things they don’t get it, they can talk about concepts, but they don’t really understand what it’s like to be in that room with that dynamic all day. Sharing experiences and just really validating each other – it took away the sense of aloneness it fills your cup, so you can come back and do it again*…*you know that person in that room totally gets what you’re going through with one look” **(Participant 13, PICU)***.

##### Respect as a Professional

Although some participants felt there was adequate acknowledgment from their peers, management, and the organization, many referenced challenges with feeling respected as a professional. Participants reported feeling disrespected by patient families, being treated like they were regarded as more of a resource than a person/professional at the level of the organization as well as not feeling valued by society in general for the work they do. “… *you see people retire and (the organization) was their life and they literally just leave and it’s like its nothing, another gear in the machine*,…*people who were these huge parts of the unit were gone and you just move on. That’s what made me realize it’s just a job” **(Participant 4, NICU)***.

##### Identity

Many participants spoke about their identity within the context of critical care nursing. They felt as though critical care nurses are unique as their role and experience differ so greatly from other areas in the hospital. As such, they require different levels of support, and they should not be expected to follow the same policies or guidelines that are applied broadly across the hospital. There was consensus that critical care work should be recognized for its uniqueness by both the organization and the public at large, *“I mean, I think we’re all biased and think that our units are the best, but I think we do exceptional work, and I think no one wants to talk about us. I think we do amazing things, but no one wants to talk about us because we’re not oncology we’re not the sellable unit, nobody wants to hear about the kid that’s in ICU it’s a parent’s worst nightmare, we’re not commercial material” **(Participant 13, PICU)***.

Many brought up how the practice of floating (a resource management strategy where nurses have to work outside their area of expertise) undermines their highly specialized skillset; “…*when we’re floating a lot, I know that, for me I’ve never worked on the Ward. I was born and raised an ICU nurse, so for me going in and floating the wards*…*I knew I was going to do it every other shift. I think (that) also contributes to burnout” **(Participant 9, PICU)***.

Some spoke about the discordance between their experience as a nurse and the “brand” of the organization, or the “nurses are heroes” trope being touted in the media, especially during the COVID-19 pandemic. “*I’m not going to lie though, a 1% raise is a bit of a slap in the face this year*… *I mean ‘you’re all heroes,’ but here’s 10 cents, you know?” **(Participant 4, NICU)***.

*“The superhero stuff, none of us buy into it, it’s for the public*” ***(Participant 13, PICU)***.

##### Quality of Work-Life Spillover

The link between well-being at work and well-being at home was identified, *“I think, because if you aren’t enjoying the work you do, then that’s going to filter in when you’re not at work as well and you’re going to dread coming into work and be thinking about it when you get home from work. If it’s a negative experience and then, when you come to work and you’re dreading it already I don’t think you’re in the right mindset. So I think if you like the work that you’re doing then overall you’ll feel better” **(Participant 18, PICU)***.

#### Work Engagement

Within the theme of work engagement three subthemes were identified: the work itself; investment in growth and development; and finding meaning in the work.

##### The Work Itself

Participants found strengths to be in the variety of the work, the challenge of the patient acuity, the collaboration with the interprofessional team, and the opportunity to engage in the most cutting-edge evidence-based practice. “…*first of all I love the variety of what I see, and I find that not every day is the same, which keeps me eager to work because I don’t know what my day is going to be like*” **(Participant 10, NICU)**.

Challenges highlighted included the gravity of the work (dealing with morbidity and mortality regularly), the pace and pressure of the work, and resource issues such as poor staffing ratios. “… *it’s kind of just expected that you’re doing it and then everybody knows that it’s inappropriate, but you’re stuck, because there’s a nursing shortage*…” **(Participant 11, NICU)**.

##### Investment in Growth and Development

Many participants felt that there were multiple opportunities for growth and development offered through their work, which contributed to their satisfaction at work and mitigated burnout. However, a few reported feeling as though opportunities beget opportunities, which resulted in the organization seeming to invest in the same cohort of people. “…*they kind of come to you, the more you branch, and I find if you’re working harder that kind of gets noticed and then opportunities present themselves within that”* **(Participant 12, CCCU**). One stated, “*I feel like there’s room for me to grow”* **(Participant 10, NICU).** Another participant spoke about the need to focus on development for those who stay at the bedside, “*Those people that stick around and teach the next generation*…*I think those people should be valued and given opportunities*” **(Participant 13, PICU)**.

##### Finding Meaning in the Work

The meaning of the work was highlighted as a major factor when it comes to feeling fulfilled and avoiding burnout. *“I think it’s a lot of fulfillment with feeling accomplished in improving a patient’s condition. So even if it’s just getting a baby to start feeds, or going up on feed successfully, or weaning ventilator settings successfully, or getting a baby out to hold with a family, I think the little things are what brings me the most fulfillment and wanting to come back” **(Participant 11, CCCU)***. Inversely, many participants spoke of the struggle when they could not find meaning in the work due to ethical dilemmas, chronicity of patients, or not feeling like they could provide their best level of care due to issues with team dynamics, the patients’ family, or availability of resources.

#### Self-Care

Within the theme of self-care, two subthemes were identified: Preparation and recovery, and separation as preservation.

##### Preparation and Recovery

Most participants referenced both proactive (routinely exercising, engaging in hobbies, formal therapy) and reactive (treating themselves, seeing friends) self-care techniques for mitigating burnout. “…*I usually make plans to either hang out with friends or go somewhere with family member. I try to create social events to look forward to*…*just do things for myself so I’ll read a book or I’ll watch a show” **(Participant 11, NICU)***. When feeling drained, the self-care goal was a restoration of balance, “*I like exercising, treating myself, whether it be going out for a coffee or something*…*finding balance” **(Participant 15, CCCU)***.

##### Separation as Preservation

Many participants brought up the necessity of having separation from work, both physically and mentally, as a protective factor. Physical separation included getting their entitled breaks during work, the benefits of the commute home to decompress, taking vacation time (paid time off), and the rotation out of stressful assignments. Mental separation included not engaging in work (checking emails, doing e-learning, etc.) on days off, and the concept of mentally leaving work at work in order to fully be present for other aspects of life. One participant shared, “*I took a personal day the next day because, I just felt like I had had 4 days with that child and they died on my fourth shift.*…*I felt like I needed some time because, I know that if I were to have come back then, I wouldn’t have been giving the next assignment my full self” **(Participant 10, NICU)***.

### Participants’ Recommendations for Improvements

Throughout the interviews, participants were asked to share ideas to address issues they brought forward as contributing to burnout in their workplace. Participants were eager to provide ideas for opportunities to reduce the negative workplace experiences and enhance experiences that promote well-being. These recommendations, from the participants themselves, are summarized in Table 1 according to which of the subthemes listed above that they address.

**TABLE 1:** Recommendations for Nursing Unit and Organizational Leadership.

**The work itself, respect as a professional, and finding meaning**
Intentional debriefs throughout complex cases to reduce the spread of false or unproductive info
Ongoing transparency around unit level and organizational level decision making that impacts frontline staff
Swift and transparent addressing of unit-based issues by management, particularly when it comes to staff safety/respect issues with patients and families
Regular debriefing with support from a bioethics team/consultation both during and after difficult cases
Thoughtful patient assignments taking into account the nurses’ previous shift experience
Transparency in assignment selection
**Benefits and compensation**
Proper compensation for extra hours worked (particularly for charge/clinical support team members who regularly leave late)
Focus on fostering unit level relationships (among nurses as well as among nurses and the interdisciplinary team)
Recognition of critical care as a unique group of nurses when it comes to compensation, for example, providing increased compensation, more mental health days, formal mental health check-ins
Initiate compensation based retention strategies for nurses in order to keep talent within the organization
**Separation as preservation**
Ensuring nurses get breaks during their shifts
Ensure staffing levels are sufficient so there is a reduced need for staff to work overtime.
Provide the opportunity for, and reduce a culture of guilt around, taking mental health days
Make designated mental health days 12 h instead of 8 h in order to benefit the frontline staff who have to most exposure to stress/trauma
Ability to ask for an assignment change or break from a specific assignment
Provide an on-site 24/h wellness space where nurses can take a break when needed
**Identity**
Ensure nurses only float to other critical care areas rather than throughout the hospital
Implement a resource nursing team to assist with staffing needs throughout the hospital
Acknowledge that critical care work and workflow is different from other areas of the hospital by ensuring consultation with critical care nurses prior to implementation of hospital-wide policies and interventions
**Investment in growth and development**
Provide opportunities for task rotation (e.g., opportunities to engage in project work)
Provide opportunities for advanced training at the bedside (e.g., certify on Continuous Renal Replacement Therapy (CRRT) and Extracorporeal Membrane Oxygenation (ECMO)
Provide opportunities for professional development and advancement

## Discussion

Throughout these 18 interviews the key themes were the experience and identification of burnout, quality of work-life, work engagement, and self-care. Consistent with the Theory of Reasoned Action and the pathway to burnout, factors of the work environment (quality of work-life) seemed to influence work attitudes (work engagement), which, subsequently influenced work outcomes (burnout). It became clear early in the analysis that the experience of burnout was common in pediatric critical care nurses as has been previously noted in the literature (11, 17). The emphasis on burnout being elusive to self-identification is a strong reminder of the need to check in regularly with teammates and colleagues, both formally and informally, to facilitate early identification of burnout. Suggestions from the literature to facilitate identification of burnout include: manager check-ins, peer support programs, Employee Assistance Programs, and Spiritual Care programs, to name a few (24, 25).

Although we did not directly aim to explore self-care-based interventions, many participants identified both preventative and restorative self-care strategies that they employ to mitigate burnout. Although not addressed by the pathway in the Theory of Reasoned Action, self-care seems to be an external strategy that participants used to mitigate the work outcome of burnout. Participants highlighted the need for separation from work as an important component of self-preservation. Similarly from an organizational perspective, recent literature has highlighted that leadership can support employee self-care by modeling good work-life balance and healthy boundaries with work, including taking paid time off, leaving work on time, and avoiding being constantly engaging with work outside of work hours/emergencies (25).

According to the Theory of Reasoned Action, work engagement is influenced by the work environment and influences work outcomes, such as burnout (14). When it comes to the theme of work engagement, Herzberg (26) describes how to motivate an employee to do something: they must have the *opportunity* and the *ability* to do it (26). This concept came through in the nurses’ discussion of their work. Opportunities for skill development/mastery, task variety, and the diversity of the patient population and technology where all highlighted as factors that improved their work engagement and, through that, their well-being. As supported by the literature, work engagement can mediate the relationship between quality of work-life and health care support workers’ intent to stay employed (27) and that between the demands of the job and nurse burnout (28). Nurse work engagement has also been shown to impact the patients’ experience of care (29). Additionally, increased work engagement has been shown to be inversely associated with nurses’ burnout and intent to leave their current employment (30, 31). Tensions within the work stemmed from lack of transparency in planning and communication and the moral distress and ethical dilemmas shown to be inherent to intensive care work (32). Potential solutions for these issues offered by our participants and supported by the literature are: regular bioethical debriefs, care planning that is shared with the interprofessional team, and check-ins and debriefs on difficult cases to reduce the spread of misinformation (24, 33).

We also heard about concepts of identity. Nurses identified deeply with the role but struggled with not being recognized as having a unique and separate skillset from other nurses at the hospital. Nurses reported feeling undervalued due to the perceived lack of recognition that critical care nurses require unique considerations within provincial and organizational policies. Floating (having to work on a unit that is not your home/specialty unit), which is a method of hospital resource allocation, has been recognized as a patient safety issue in the Registered Nurse Safe Staffing Act of 2015, where the American Nurses Association asserts that hospitals should not require nurses to float outside of their education/training/specialty to avoid harm to both the patients and nurses (34). One solution to the challenge of floating, suggested by O’Connor and Duggan (34), is to have designated nurse resource teams that have diverse experience and have trained in several areas of the hospital (34). Particularly within the context of the COVID-19 pandemic, it has come to light that nurses are not interchangeable (35). Critical care trained nurses are an extremely valuable and limited resource with many calling for revision of RN compensation regulations as thousands of nurses leave the field while policies like Ontario’s Bill C124 caps their wage increases during an unprecedented pandemic (36). Additionally, lack of autonomy and respect by administrators was cited as a major factor in attrition in a 2013 ethnographic study of PICU nurses in British Columbia, Canada (37). Hollow praise (e.g., the empty words without action or being called ‘‘health care heroes’’) and clichéd gestures (e.g., break room ‘‘resiliency pizza’’)^1^ are not what these nurses find helpful to mitigate or prevent burnout. Alternatives could be including nurses in unit and corporate decision-making, focusing on recognition strategies that support nurses in their work (like safe staffing ratios and incentives to stay in the field), regular performance feedback, and opportunities for growth and development (24, 25, 33).

On the positive side, we heard many stories about the nurses’ identity within their team and what a protective factor their work relationships can be. The bonds built between colleagues are unique to both the context in which they are formed in and the stress they endure. Similar findings have been reported from studies on the concept referred to as “unit cohesion,” particularly in military unit bonding where it is identified as a source of resilience and a mitigator of Post-Traumatic Stress Disorder and depression (38). Group cohesion has also been shown to improve nurse satisfaction and retention (39). These distinctive bonds also support the role of context-based peer support as a frequently cited source of respite in our sample. Group cohesion can be fostered with team building activities, unit social events outside of work, and peer-to-peer support networks (24, 25).

## Limitations

This study used an explanatory qualitative approach which does not allow for generalizability of our findings. It is also limited by the location, time, and context in which the interviews were conducted. This study took place in the unique context of the COVID-19 pandemic which undoubtedly increased the workplace stress on the staff during this time (40, 41). However, this context may have allowed the nuances of workplace well-being to be further illuminated and thus brought forward in our results. There is possible bias in participant responses due to perceived social desirability, despite assured anonymity of how the findings would be shared (42). As well, potential bias may have been introduced by the order in which concepts were presented from the semi-structured interview guide. Participants self-selected to participate in both phases of the study; those with the most extreme feelings may be over-represented (42). Finally, although reflexivity was practiced throughout the study, the PI’s identity as a pediatric critical care nurse who cares about the well-being of fellow pediatric critical care nurses may have shaped the findings.

## Future Implications

This was a particularly opportune time to conduct research on nurse well-being. As we proceed through the later phases of the COVID-19 pandemic, mitigation of nurse burnout is more important than ever (25). Evidence-based recommendations, such as the results presented in this study, should be disseminated to nursing leadership, hospital administration, professional organizations, and government officials. Further research is needed to explore how pediatric nurse quality of work-life and work engagement impacts their experience of burnout including implementing and evaluating burnout interventions. By using data gathered directly from the nurses, interventions can be crafted to not only mitigate burnout, but prevent it from occurring.

## Conclusion

This study expands our understanding of the experience of burnout and contributing factors in the understudied population of pediatric critical care nurses. Pediatric critical care nurses carry the trauma of what is unthinkable to most, and they do it with steady hands, expert skill, and compassion. As nurse researchers we owe them our time and our interpretation to voice their concerns and share their solutions with the organization and higher levels of leadership. Further research is needed on the outcomes of well-being interventions in pediatric critical care nurses. They are dynamic professionals that must be fostered in order to keep them thriving in their complex work; they deserve to be well in the process.

## Data Availability Statement

The raw data supporting the conclusions of this article will be made available by the authors, without undue reservation.

## Ethics Statement

The studies involving human participants were reviewed and approved by the Research Ethics Board at The Hospital for Sick Children in Toronto, Canada (REB #1000072502). It is also approved by the University of Toronto. The patients/participants provided their written informed consent to participate in this study.

## Author Contributions

LB was involved in the study design, data collection, data analysis, data interpretation, and drafting and finalizing the manuscript. WB and KC were involved in data interpretation, and substantively revised the manuscript for important intellectual content. KW was involved in the study design, data interpretation, and substantively revised the manuscript for important intellectual content. All authors read and approved the final manuscript and agreed both to be personally accountable for their own contributions and to ensure that questions related to the accuracy or integrity of any part of the work are appropriately investigated, resolved, and the resolution documented in the literature.

## Conflict of Interest

The authors declare that the research was conducted in the absence of any commercial or financial relationships that could be construed as a potential conflict of interest.

## Publisher’s Note

All claims expressed in this article are solely those of the authors and do not necessarily represent those of their affiliated organizations, or those of the publisher, the editors and the reviewers. Any product that may be evaluated in this article, or claim that may be made by its manufacturer, is not guaranteed or endorsed by the publisher.

## Footnotes

1 Resiliency pizza refers to the pizza given to nurses as a “thank you” from hospital administration for working under less-than-ideal working conditions. The gesture is often received as an insult to those working at the bedside.

## Abbreviations

PICU: Pediatric Intensive Care Unit
CCCU: Cardiac Critical Care Unit
NICU: Neonatal Intensive Care Unit.
