# Supplementary material for: Exploring Pediatric Nurses’ Perspectives on Their Work Environment, Work Attitudes, and Experience of Burnout: What Really Matters?
Source: Front Pediatr. 2022 Mar 17;10:851001. doi: 10.3389/fped.2022.851001 (PMC8968655; doi:10.3389/fped.2022.851001)
Supplement: Supplementary file 1 [file Data_Sheet_1.docx]

**Appendix A**

| **WE CARE Semi-structured Interview Guide** | | |
| --- | --- | --- |
| **Domain** | **Question(s)** | **Prompts** |
| **Quality of Work-Life** | Can you tell me about the aspects of your job that keep you coming back every day?  What things does the unit/hospital that shows you they care about you and your professional development?  Why do you think the quality of work-life has such a strong relationship to burnout?  What aspects of your job do you like least? | Are your needs being met? Physical, financial, emotional, etc)  How do your experiences at work contribute to your overall well-being?  What could they do more of to make you feel appreciated and valued? What would you change, if you could? |
| **Work Engagement** | Can you give an example of a time when you felt very engaged at work?  How about a time that you did not feel engaged at work?  How does not feeling engaged in your work contribute to your feelings of being burned out? | What was going on? Who was involved? How did you feel?  What was going on? Who was involved? How did you feel?  Does it have a big impact? Little impact? No impact?  When you are feeling burned out and not engaged in your work, what helps turn this around? How does/can the organization help? What personal things do you do to help? |
| **Burnout** | Have you experienced burnout at work? If so, what does it feel like?  What is the unit or organization doing to prevent you from getting burned out? What resources are in place that you use?  What resources do you not use/avoid?  What could the unit or organization start or do more of to prevent you from getting burned out? | What were the contributing factors? Were they all related to work or were some factors external to work? What were your symptoms? How did it impact your day to day life? (exhaustion, low mood, ‘doing the bare minimum’)  What could the unit or organization stop/ do less of to prevent you from getting burned out?  Why do you use or not use specific resources? (ex: hard to access? No time? Unsure of how to enroll?) |
| **Closing** | Do you have anything else you would like us to know about how the organization can best support you in being successful and happy at work? |  |

**When the conversation has slowed:**

1. Ask for any last closing comments that the participant feels is important and were not addressed or emphasized.
2. Ensure the participant has the contact information of the study team should they have any follow up questions or concerns.

Thank the participant for their time and participation, reiterate that their information and audio recordings will be de-identified. Let them know they will have access to the results of the overall study once it is complete and submitted for presentation and publication
